# The role of 5-HT_2C_ receptors in touchscreen visual reversal learning in the rat: a cross-site study

**DOI:** 10.1007/s00213-015-3963-5

**Published:** 2015-05-26

**Authors:** J. Alsiö, S. R. O. Nilsson, F. Gastambide, R. A. H. Wang, S. A. Dam, A. C. Mar, M. Tricklebank, T. W. Robbins

**Affiliations:** Department of Psychology, University of Cambridge, Cambridge, CB2 3EB UK; Behavioural and Clinical Neuroscience Institute, University of Cambridge, Cambridge, CB2 3EB UK; Department of Neuroscience, Unit of Functional Neurobiology, University of Uppsala, Uppsala, SE-75124 Sweden; Lilly Centre for Cognitive Neuroscience, Eli Lilly & Co. Ltd., Erl Wood Manor, Windlesham, GU20 6PH UK

**Keywords:** Reversal learning, SB 242084, 5-HT_2C_ receptor, Orbitofrontal cortex, Rat

## Abstract

**Rationale:**

Reversal learning requires associative learning and executive functioning to suppress non-adaptive responding. Reversal-learning deficits are observed in e.g. schizophrenia and obsessive-compulsive disorder and implicate neural circuitry including the orbitofrontal cortex (OFC). Serotonergic function has been strongly linked to visual reversal learning in humans and experimental animals but less is known about which receptor subtypes are involved.

**Objectives:**

The objectives of the study were to test the effects of systemic and intra-OFC 5-HT_2C_-receptor antagonism on visual reversal learning in rats and assess the psychological mechanisms underlying these effects within novel touchscreen paradigms.

**Methods:**

In experiments 1–2, we used a novel 3-stimulus task to investigate the effects of 5-HT_2C_-receptor antagonism through SB 242084 (0.1, 0.5 and 1.0 mg/kg i.p.) cross-site. Experiment 3 assessed the effects of SB 242084 in 2-choice reversal learning. In experiment 4, we validated a novel touchscreen serial visual reversal task suitable for neuropharmacological microinfusions by baclofen-/muscimol-induced OFC inactivation. In experiment 5, we tested the effect of intra-OFC SB 242084 (1.0 or 3.0 μg/side) on performance in this task.

**Results:**

In experiments 1–3, SB 242084 reduced early errors but increased late errors to criterion. In experiment 5, intra-OFC SB 242084 reduced early errors without increasing late errors in a reversal paradigm validated as OFC dependent (experiment 4).

**Conclusion:**

Intra-OFC 5-HT_2C_-receptor antagonism decreases perseveration in novel touchscreen reversal-learning paradigms for the rat. Systemic 5-HT_2C_-receptor antagonism additionally impairs late learning—a robust effect observed cross-site and potentially linked to impulsivity. These conclusions are discussed in terms of neural mechanisms underlying reversal learning and their relevance to psychiatric disorders.

**Electronic supplementary material:**

The online version of this article (doi:10.1007/s00213-015-3963-5) contains supplementary material, which is available to authorized users.

## Introduction

Purposeful goal-directed behaviour requires flexible responding to altered reinforcement contingences. In experimental animals, such flexible responding is commonly assessed using an appetitive, operant reversal-learning paradigm in which initially learned reward contingencies are switched. In order to successfully adapt and maximise the amount of reward earned, subjects must not only learn to suppress selection of the previously rewarded responses but also to learn a novel association and to choose the previously unrewarded (but now rewarded) option. In humans, impaired cognitive flexibility has been observed across tasks in schizophrenic patients (Tyson et al. 2004; Jazbec et al. 2007; Murray et al. 2008; Ceaser et al. 2008; Pantelis et al. 2009), but reversal-learning deficits stand out as a core feature of first-episode psychosis (Leeson et al. 2009). Despite a growing literature on such impairment, the study of cognitive function in the evaluation and development of novel antipsychotic treatments has only recently received due focus (Moore et al. 2013).

Much evidence implicates circuitry including the orbitofrontal cortex (OFC), ventral (NAc) and dorsal striatum (DStr) and the amygdala in reversal learning (Clark et al. 2004). In humans, the OFC has been shown to be activated in fMRI studies of reversal learning (Hampshire and Owen 2006; Chamberlain et al. 2008). OFC-lesioned marmosets show selective reversal deficits in an intra-dimensional/extra-dimensional set-shifting task (Dias et al. 1996). OFC lesion or inactivation has also repeatedly been found to impair simple or serial reversal learning in rodents (Bussey et al. 1997; Schoenbaum et al. 2002; Chudasama and Robbins 2003; McAlonan and Brown 2003; Kim and Ragozzino 2005; Ghods-Sharifi et al. 2008; Bissonette et al. 2008; Burke et al. 2009; Graybeal et al. 2011).

Reversal learning depends on serotonin (5-hydroxytrypamine or 5-HT) signalling. It is impaired following acute tryptophan depletion in healthy human volunteers (Park et al. 1994), and central 5-HT depletion in experimental animals retards go/no-go reversal (Masaki et al. 2006), bowl-digging reversal (Lapiz-Bluhm et al. 2009) and probabilistic reversal learning in the rat (Bari et al. 2010). Increasing 5-HT levels through pharmacological or genetic inactivation of the 5-HT transporter, in contrast, improves performance in visual reversal (Brigman et al. 2010) and serial spatial reversal learning (Barlow et al. 2015). These effects may be related to altered activity at 5-HT receptors specifically within the OFC. OFC-selective 5-HT depletion retards serial visual reversal learning in the marmoset (Clarke et al. 2004; Clarke et al. 2005; Clarke et al. 2007) while OFC 5-HT markers have been found to correlate with reversal performance in the rat (Masaki et al. 2006; Barlow et al. 2015). Furthermore, systemic (Boulougouris et al. 2008; Nilsson et al. 2012) or OFC-specific (Boulougouris and Robbins 2010) antagonism of the 5-HT_2C_ receptor (5-HT_2C_R) through SB 242084 can improve spatial reversal-learning performance in rodents, whereas blockade of the 5-HT_2A_ receptor, in contrast, impairs reversal learning.

These findings may be relevant for reversal-learning impairment associated with schizophrenia (Leeson et al. 2009): 5-HT levels in schizophrenic patients have been shown to correlate with cortical atrophy (Van Kammen et al. 1985), severity of cognitive impairment (Powchik et al. 1998), hypofrontality during tests of cognitive flexibility (Weinberger et al. 1988) and poor long-term clinical outcome (Wieselgren and Lindström 1998). Furthermore, prefrontal cortical aberrations in 5-HT_2C_R pre-mRNA editing (Sodhi et al. 2001), 5-HT_2C_R mRNA levels (Castensson et al. 2005) and 5-HT_2_R binding (Arora and Meltzer 1991; Powchik et al. 1998) have been reported in schizophrenic patients. Smaller OFC volume has also been observed in schizophrenic patients (Nakamura et al. 2008).

However, tests of reversal learning in human patients use visual cues whereas preclinical testing in rodents generally employs olfactory or spatial learning. Thus, touchscreen visual reversal-learning paradigms have been developed for cognitive testing that have been shown to be sensitive to OFC-lesioning in both rats and mice (Bussey et al. 1997; Chudasama and Robbins 2003; Graybeal et al. 2011) and genetic manipulations in the mouse (Brigman et al. 2010; Nithianantharajah et al. 2012).

The aim of the current study was to investigate the effects of the 5-HT_2C_R antagonist, SB 242084, administered systemically or intra-OFC during reversal learning using a series of novel touchscreen tasks. In experiments 1 and 2, we developed a 3-stimulus reversal paradigm for the rat previously used to test object reversal learning in primates (Jentsch et al. 2002; Lee et al. 2007) to assess the dose-dependent effects of SB 242084. This specific paradigm was chosen to illuminate potential perseverative reversal deficits: after contingency shifts (reversal) in this paradigm, the presence of two CS− response options allows the separation of errors according to perseverative responses (at the previous CS+) and non-perseverative responses (at the ‘constant CS−’). To demonstrate the robustness and reliability of the drug effects within the 3-stimulus reversal paradigm, the initial findings conducted at an academic site (Cambridge, UK) were replicated and extended by an industrial partner (Lilly, UK) as part of the NEWMEDS initiative (http://www.newmeds-europe.com). Experiment 3 investigated the dose-dependent effects of systemic 5-HT_2C_R antagonism through SB 242084 on a well-established two-choice visual reversal-learning paradigm (Mar et al. 2013). In experiment 4, in order to investigate the neuroanatomical substrate of the observed effects, we developed a novel serial reversal-learning task suitable for use in subregion-specific pharmacological studies exploring perseverative behaviour, and confirmed an OFC dependency on task performance through baclofen/muscimol inactivation. In experiment 5, we assessed the effect of intra-OFC 5-HT_2C_R antagonism on reversal learning by site-specific SB 242084 infusions in this novel task.

## Methods and materials

### Animals

Experiment 1 was performed at the academic partner (University of Cambridge) and used 25 male Lister hooded rats (Charles River, UK). Experiment 2 and experiment 3 were run at the industrial partner (Eli Lilly) and used separate groups of 46 male Lister hooded rats (Harlan, UK). Animals in experiments 1, 2 and 3 were housed in groups of four. Animals who failed to complete any stage of experiments 1–3 were excluded from the analysis; see ‘Experimental design and statistical analyses’, below. Following surgical implantation of guide cannulae, the animals (Charles River, UK) in experiment 4 (*N* = 10) and experiment 5 (*N* = 13) were single housed (experiments run at University of Cambridge). Animals were food-deprived with ad libitum access to water, and their body weights were maintained at about 85 % of their free-feeding weight. Animals were fed each day 1 h after testing. Animals were weighed weekly or each day during drug administration. Rats were maintained under a 12-h light/dark cycle, with lights on at 7 PM (academic partner) or 7 AM (industrial partner). The experiments were conducted in accordance with the UK Animals (Scientific Procedures) Act 1986.

### Drugs

SB 242084 (Eli Lilly, Indianapolis, IN, USA) was initially dissolved in PEG400 (Fisher Scientific, Loughborough, UK) at 20 % of the final required volume, which was then made up by 10 % (*w*/*v*) hydroxypropyl-beta-cyclodextrin (Sigma-Aldrich, Poole, UK) in saline. Aliquots were frozen at −80 °C in the quantities required for each test day. For systemic treatment (experiments 1–3), SB 242084 was administered intraperitoneally (i.p.) at doses of 0 (vehicle), 0.1, 0.5 or 1.0 mg/kg in a volume of 1 ml/kg 20 min prior to testing. For intra-OFC microinfusions in experiment 5, SB 242084 was administered at 0 (vehicle), 1 or 3 μg/hemisphere immediately before testing. Baclofen hydrochloride (Sigma-Aldrich) and muscimol hydrobromide (Sigma-Aldrich) were dissolved separately in sterile saline and prepared as a cocktail with each drug at a final concentration of 1.0 mM (Zeeb et al. 2010).

### Experiments 1–3: 2- and 3-stimulus reversal learning

A comparison of the different protocol parameters in experiments 1–3 is shown in Table 2 and Supplementary Fig. 1. Rats were pretrained to respond at a touchscreen in a behavioural chamber (Med Associates, Georgia, VT, USA) to receive 45 mg sucrose reward pellets (Sandown Scientific, Middlesex, UK). Full details of the pretraining procedure and of the apparatus are provided in the Supplementary Material.

#### 3-Stimulus discrimination and reversal learning

For experiment 1, a rodent 3-stimulus reversal task was employed, based on prior, unpublished development work at the University of Cambridge (Mar et al. 2012). Following trial initiation, three different stimuli (one stimulus designated as CS+, two stimuli designated as CS − s; Table 1) were presented in three different response windows on the touchscreen. The six possible spatial stimulus configurations occurred equal number of times over every 30 trials but the same configuration never recurred for more than two consecutive trials. If the animal touched the CS+, all stimuli were removed from the touchscreen and a reward pellet was delivered. If the animal touched a CS−, all stimuli were removed, the houselight was illuminated for a 5-s time-out period and an incorrect response was recorded. After the animal collected the reward pellet after a correct trial or after the 5-s time-out had elapsed following an incorrect trial, a 5-s ITI was initiated. After the ITI had elapsed, the magazine-light began flashing at 1 Hz and a new trial started when the animal nose poked in the magazine (see Supplementary Fig. 1). The session ended after 45 min or 100 correct trials. The criterion for visual discrimination learning and all subsequent tests was ≥9 correct responses over 10 trials twice in one session using a rolling trial count. When criterion was reached, animals were challenged with a reversal on the following day.

**Table 1:** Comparison of experimental parameters

	Experiments 1 and 2	Experiment 3	Experiment 4 and 5
	3-stimulus reversal	2-stimulus reversal	2-stimulus serial reversal
Trials per session	Unlimited	100	250
Correct per session	100	100	150
Incorrect per session	Unlimited	100	250
Omissions	NA	>10 s	>10 s
Time-out	5 s	5 s	5 s
Design	Between-subjects	Between-subjects	Within-subjects
Criterion	≥9 correct over 10 trials twice in one session	≥9 correct over 10 trials twice in one session	≥24 correct over 30 trials

Experiment 1: vehicle *N* = 16, 1.0 mg/kg *N* = 9. Experiment 2: vehicle *N* = 10, 0.1 mg/kg *N* = 7, 0.5 mg/kg *N* = 7, 1.0 mg/kg *N* = 9. Experiment 3: vehicle *N* = 12, 0.1 mg/kg *N* = 11, 0.5 mg/kg *N* = 12, 1.0 mg/kg *N* = 11. Experiment 4 *N* = 6. Experiment 5 *N* = 10

Experiment 2 was designed to replicate the procedure from experiment 1 but at the laboratory of the industrial partner (Eli Lilly) rather than by the academic partner (University of Cambridge). However, stimuli reward contingencies were not counterbalanced in experiment 2 but counterbalanced in experiment 1.

#### 2-Stimulus discrimination and reversal learning

The procedure of experiment 3 was adapted to parallel previous protocols investigating 5-HT_2C_R function and reversal learning (Nilsson et al. 2012; Boulougouris et al. 2008; Boulougouris and Robbins 2010). In the test phase, animals were required to touch the stimulus within 10 s and the number of trials was limited to 100 per session. After trial initiation, two stimuli (one stimulus designated as CS+ and one stimulus designated CS−) were presented in the two response windows. If the animal touched the CS+, all stimuli were removed and a pellet reward was delivered. If the animal touched the CS−, all stimuli were removed, the houselight was illuminated for a 5-s time-out period and an incorrect response was recorded. If the animal failed to respond within 10 s, all stimuli were removed from the screen, the 5-s ITI was initiated and an omission was recorded. After the animal collected the reward pellet after a correct trial or after the 5-s time-out had elapsed following an incorrect trial, a 5-s ITI was initiated. After the ITI had elapsed, the magazine-light began flashing at 1 Hz. A new trial started when the animal nose poked into the magazine (see Supplementary Fig. 1). The session ended after 45 min or 100 trials. The criterion for discrimination and reversal learning was 9 correct responses over 10 trials twice in one session using a rolling trial count. When criterion was reached, animals were challenged with a reversal on the next day.

### Experiments 4–5: 2-stimulus serial visual reversal learning

This paradigm was designed to allow rapid serial reversal learning in the rat with consistent and high levels of perseverative behaviour after each contingency shift. Task parameters (e.g. stimuli, criteria for learning and the number of retention sessions between reversals) were defined and optimised in an initial cohort of rats that did not receive intra-cerebral infusions (data not shown). The resulting procedure, where each reversal phase typically took 3 days, is described in the Supplementary Material and briefly summarised below.

#### 2-Stimulus serial discrimination reversal learning

Rats were trained to respond at two stimuli simultaneously presented on the screen, similar to experiment 3, above. Thus, one stimulus (CS+) was associated with reward and the other stimulus (CS−) with a houselight-signalled time-out of 5 s. However, stimuli were horizontal and vertical bars, to ensure rapid discrimination learning, and trials were initiated by responding at a ‘start box’ at the bottom centre of the screen. The start-box procedure was used to ensure a central position of the animal before the choice phase, based on prior development work by A.C.M. using similar touchscreen procedures (Mar et al., 2012). No masks were used for this paradigm.

The session ended after 150 rewards, 250 trials or 60 min, whichever occurred first (Table 2). The ITI was set to 5 s and the limited hold (stimulus presentation time and response window) was set to 10 s (see Supplementary Fig. 1). Criterion for discrimination learning was set to 24 correct in a running window of 30 trials. Once acquired, rats were given a retention session using the same reward contingencies to confirm that the rats had acquired the discrimination.

**Table 2:**
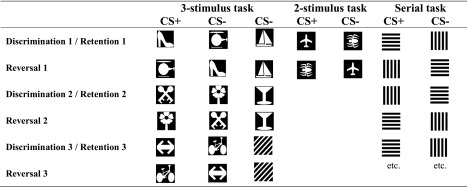
Stimulus-reward contingencies

Stimulus-reinforcement contingencies in 2-stimulus reversal learning (Experiment 3), 3-stimulus reversal learning (Experiment 1 and 2) and serial reversal learning (Experiment 4 and 5).

Following the discrimination and a retention session, the contingencies reversed and the rats were required to respond to the previous CS− until they reached the reversal criterion (24/30). A retention session was included before each reversal and after criterion was met. Additional reversals were performed until the rats were able to reach the criterion within three daily sessions.

#### Surgery

The rats were surgically implanted with 22-GA guide cannulae (PlasticsOne, Roanoke, VA, USA) under isoflurane anaesthesia (anaesthesia was induced at 4 % and maintained at 2 % isoflurane). The tooth bar was set to −3.3 mm for flat skull position. Targets for lateral OFC guides were AP +3.7, ML ±2.5 (from bregma) and DV −1.7 mm (from dura). The guide cannulae were secured to the skull using four metal screws and dental cement. Obturators that ended flush with the guide cannulae were inserted and protected with a dust cap.

#### Microinfusions

After recovery from surgery (≥7 days), rats received a retention session and were then reversed until criterion (followed by another retention session) without drug infusion to verify fast and stable serial reversal performance. During this baseline reversal, rats were habituated to the infusion procedure and received mock and vehicle infusions. Injectors from PlasticsOne (28-GA) were extended 2 mm below the guide for OFC infusions (−3.7 mm from dura). Infusions were performed in a volume of 0.5 μl over 2 min. The injector was left in place for 1 min before and after infusion. During the infusion procedure, the rats were gently restrained or allowed to freely move on the lap of the experimenter.

Following the baseline reversal, rats received intracerebral infusions of baclofen/muscimol mix (experiment 4) or SB 242084 (experiment 5) across reversals according to a within-subject, cross-over/Latin-square design. Microinfusions were given each day of the reversal, i.e. from the session when contingencies first shifted to the day that the rats reached criterion on the task. Rats that reached criterion on the third day thus received three infusions on consecutive days during that reversal. Retention sessions (no infusions) were included the day after criterion was met and again before the next reversal started. Rats typically had 2 days without testing between these retention sessions (i.e. a full reversal with retention sessions and break was 7 days, during which the rats typically received three infusions). In a few instances, rats did not reach criterion during a retention session; these rats received a second retention session on the following day and invariably reached criterion on this additional session.

#### Histology

At the end of the experiments, animals were given a lethal dose of sodium pentobarbitone and perfused transcardially with 0.01 M PBS followed by 4 % paraformaldehyde. The brains were removed, postfixed in 4 % paraformaldehyde for 24 h and preserved in 30 % sucrose in 0.01 M PBS overnight. Coronal sections (60 μm) were stained with cresyl violet and used to verify injector-tip placement inside the lateral OFC according to a standard rat brain atlas (Paxinos and Watson 1998).

### Experimental design and statistical analyses

#### Experiments 1–3

These experiments employed between-subject designs. Experiments 1 and 2 used a serial design with new stimulus triplets presented in each new discrimination phase (Table 1). After completing an initial three-choice discrimination drug free, animals were matched for trials to criterion and assigned to a drug dose for the first reversal. Animals subsequently completed two more three-choice visual discriminations followed by reversals. Animals were dosed in reversal 1, reversal 2 and visual discrimination 3. Animals completed visual discrimination 1, visual discrimination 2 and reversal 3 drug free.

In Experiment 3, animals initially completed a two-choice discrimination drug free and were subsequently matched for trials to criterion and assigned to a drug dose for reversal testing (Table 2). The stimuli used in experiment 1 were based on previous reports showing that animals have minimal spontaneous visual biases for this stimulus pair (Bussey et al. 2008).

The primary dependent variables for experiments 1, 2 and 3 were trials and errors to criterion. In addition, we analysed correct responses to criterion as well as response latency and pellet-retrieval latency. Latency data from experiment 1 were lost due to a computer malfunction. In the 3-stimulus discrimination (experiments 1 and 2), incorrect responses were further divided into responses towards the previous CS+ (‘previous CS+’ errors) and responses towards the constant CS− (constant CS− errors). In the reversal phases of experiment 1–3, incorrect responses were additionally coded as early errors and late errors corresponding to before and after animals had reached random responding. Thus, early errors were the number of incorrect responses made before achieving 33 % (≥3 correct responses over 10 trials) twice in the 3-stimulus paradigm and the number of incorrect responses made before achieving 50 % (≥5 correct responses over 10 trials) twice in the 2-stimulus paradigm. Late errors corresponded to errors made after reaching 33 and 50 %, respectively. The data for the three procedures of experiments 1 and 2 where SB 242084 was administered were analysed by 3 (within-subjects: phase) × 4 (between-subjects: drug dose) mixed ANOVAs. The data for the reversal phase in experiment 3, as well as the third, drug-free reversal phase test in experiments 1 and 2, were analysed by one-way between-subjects ANOVAs with drug-dose group as the independent variable. Significant interactions were followed by LSD post-hoc comparisons versus vehicle.

Animals who failed to complete any stage of experiments 1–3 were excluded from the analysis (experiment 1: vehicle *N* = 2, 1.0 mg/kg *N* = 2; experiment 2: vehicle *N* = 1, 0.1 mg/kg *N* = 3, 0.5 mg/kg *N* = 3, 1.0 mg/kg *N* = 3; experiment 3: vehicle *N* = 0, 0.1 mg/kg *N* = 1, 0.5 mg/kg *N* = 0, 1.0 mg/kg *N* = 1).

#### Experiments 4–5

These experiments employed within-subjects designs. Data from each reversal were collapsed over days. Trial outcomes were next coded as perseverative, random or learning depending on performance over 30 trials and based on binomial distribution probabilities. Thus, any 30-trial bin in which the rat displayed a significant bias towards the previously correct stimulus (<11 correct) was coded as perseverative, whereas any 30-trial bin in which the rat displayed a significant bias towards the currently correct stimulus (>19 correct) was coded as learning. Bins were coded as perseverative or learning wherever they occurred during the session, meaning that rats technically could shift multiple times between perseverative and random, and random and learning phases. In experiments 1–5, data from after criterion was reached were excluded from analysis.

The primary dependent variables were trials, errors and omissions in each phase (note that omissions only occurred if the animals actively initiated a trial by touching the start box). Latencies to respond at the stimuli (after initiating a trial) and to collect reward pellets were additionally analysed. Analysis of the data from the first cohort of rats (data not shown) suggested that square-root transformation produced normal distribution of scores for trials, incorrect responses and omissions. Transformed data were analysed using two-way repeated measures ANOVA, in a 2 (baclofen/muscimol dose) × 3 (phase) or in a 3 (SB 242084 dose) × 3 (phase) design. Planned comparisons were performed for the effects of both baclofen/muscimol inactivation and of SB 242084 on perseverative performance. Percent correct responses on the first day of reversal was analysed as an alternative measure of early, perseverative performance, using one-way repeated-measures ANOVA or paired *t*-test as appropriate. Similarly, performance in 30-trial bins were analysed for the first 300 trials using two-way repeated-measures ANOVA (Supplementary Fig. 2). For latency scores, median values for the different doses for each subject were entered into the statistical analysis. Animals that lost their cannulae during the course of the experiment (experiment 4: *N* = 3; experiment 5: *N* = 3), as well as animals with injector tips outside the lateral OFC (experiment 4: *N* = 1), were excluded from all analyses.

## Results

### Experiment 1—SB 242084 and 3-stimulus serial visual reversal learning

**Fig. 1:**
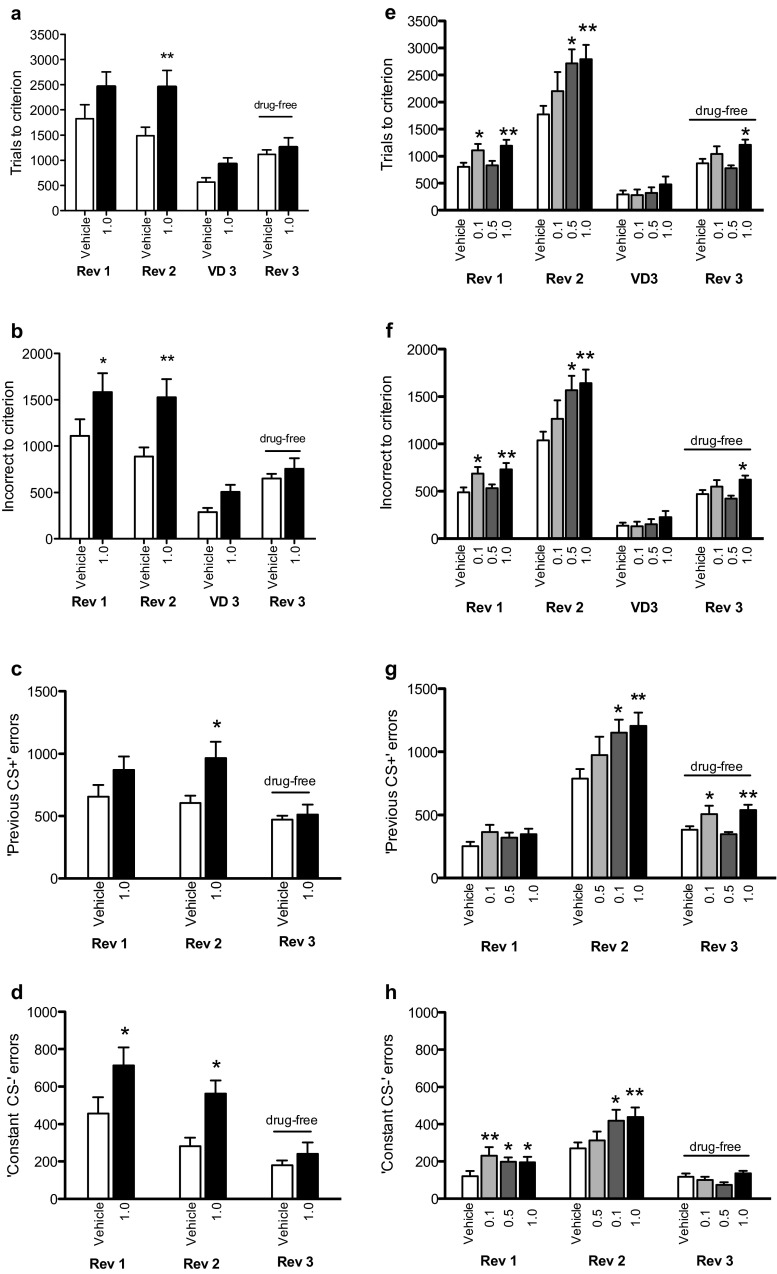
Effects of SB 242084 on 3-stimulus visual discrimination and reversal learning in the rat at the academic (experiment 1; **a**–**d**) and industrial (experiment 2; **e**–**h**) partners. In reversal learning, SB 242084 increased trials (**a**, **e**), incorrect responses (**b**, **f**) ‘previous CS+’ errors (**c**, **g**) and ‘constant CS−’ errors (**d**, **h**) to criterion at both the academic and industrial sites. Stimulus-reward contingencies were not counterbalanced in experiment 2 but counterbalanced in experiment 1. *Asterisks* denote *p* < 0.05 vs. vehicle (*p < 0.05; **p < 0.01)

SB 242084 had a main effect on trials to criterion (Fig. 1a; *F*_1,18_ = 7.662, *p* = 0.013); post hoc analysis revealed a significant impairment on the second reversal (*p* = 0.005) but not the first reversal (*p* = 0.055) or visual discrimination (*p* > 0.10).

SB 242084 also had an effect on incorrect responses to criterion (Fig. 1b; *F*_1,18_ = 8.370, *p* = 0.001), with increased number of errors in the first (*p* = 0.026) and on the second reversal (*p* = 0.003) but not discrimination learning (*p* > 0.10).

Previous CS+ errors were also affected by the SB 242084 dose (Fig. 1c; *F*_1,18_ = 5.086, *p* = 0.037), with a significant increase in the second reversal (*p* = 0.004) but not the first reversal (*p* = 0.081) or the drug-free third reversal (*p* > 0.10). SB 242084 increased constant CS− errors (Fig. 1d; main effect of drug; *F*_1,18_ = 7.679, *p* = 0.013) during both the first (*p* = 0.011) and second (*p* = 0.006) reversals.

**Fig. 2:**
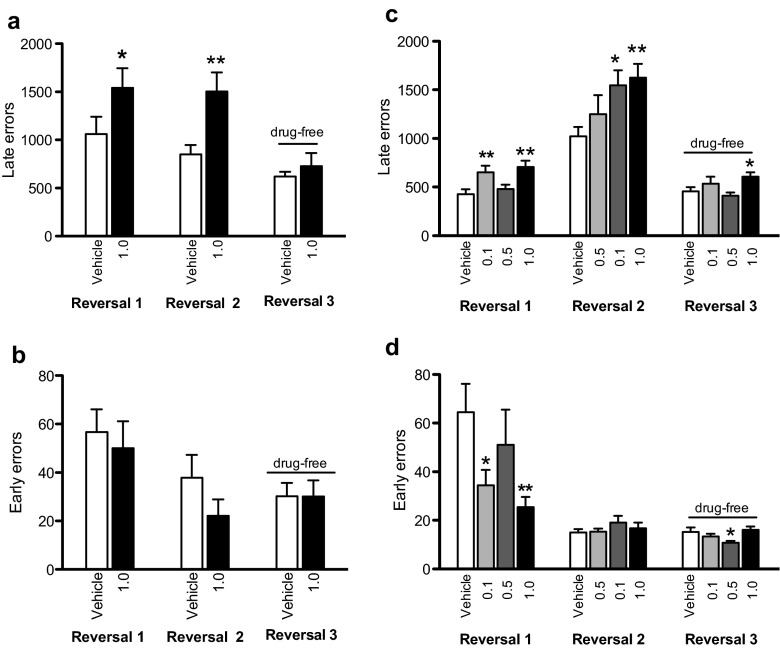
Effects of SB 242084 on early errors and late errors in 3-stimulus reversal learning at the academic (experiment 1; **a**, **b**) and the industrial (experiment 2; **c**, **d**) partners. SB 242084 increased late errors in both laboratories (**a**, **c**). SB 242084 decreased early errors but the effect reached significance only at the industrial site (**b**, **d**). Stimulus-reward contingencies were not counterbalanced in experiment 2 but counterbalanced in experiment 1. *Asterisks* denote *p* < 0.05 vs. vehicle (*p < 0.05; **p < 0.01)

SB 242084 numerically reduced early errors but the effect was not significant (Fig. 2b; *F*_1,18_ = 1.506, *p* > 0.10). In contrast, late errors were significantly affected by the drug (Fig. 2a; *F*_1,18_ = 6.774, *p* = 0.018). SB 242084 increased late errors during the first (*p* = 0.028) and the second reversal (*p* = 0.003). On the last, drug-free reversal, no differences between groups were observed (*p* > 0.10).

### Experiment 2—replication of SB 242084-induced effects on 3-stimulus visual reversal learning in a partner laboratory

SB 242084 decreased early errors but increased late errors causing an overall increase in trials and incorrect responses to criterion on reversal learning (Figs. 1 and 2). There were no effects of SB 242084 on visual discrimination learning. SB 242084 decreased stimulus response latencies in both visual discrimination and reversal learning (Table 3).

**Table 3:** Mean response latency and reinforcer retrieval latency in seconds (±SEM) in experiment 2

	Reversal 1		Reversal 2		Discrimination 3		Reversal 3^a^	
Dose	Response	Retrieval	Response	Retrieval	Response	Retrieval	Response	Retrieval
Vehicle	3.63 ± 0.30	1.84 ± 0.13	3.81 ± 0.47	1.51 ± 0.04	2.96 ± 0.23	1.40 ± 0.05	3.00 ± 0.20	1.54 ± 0.11
0.1	2.90 ± 0.09	1.61 ± 0.14*	2.81 ± 0.24*	1.59 ± 0.09	2.31 ± 0.11*	1.33 ± 0.06	3.28 ± 0.41	1.43 ± 0.05
0.5	3.26 ± 0.38	1.56 ± 0.09**	2.72 ± 0.14*	1.55 ± 0.12	2.65 ± 0.31	1.34 ± 0.08	3.73 ± 0.23	1.67 ± 0.19
1. 0	2.81 ± 0.24*	1.47 ± 0.10*	2.70 ± 0.15*	1.71 ± 0.14	2.27 ± 0.18*	1.43 ± 0.05	3.58 ± 0.31	1.49 ± 0.05

Asterisks denote significant difference from vehicle (*p < 0.05; **p < 0.01)

^a^Drug free

On trials to criterion (Fig. 1e), there was a significant main effects of test phase (*F*_2,58_ = 157.96, *p* < 0.0001) and dose (*F*_3,29_ = 5.124, *p* = 0.006) and a significant dose × phase interaction (*F*_6,58_ = 2.489, *p* = 0.033); 0.1 mg/kg (*p* = 0.035) and 1 mg/kg (*p* = 0.005) of SB 242084 increased trials to criterion in reversal one, and 0.5 mg/kg (*p* = 0.013) and 1 mg/kg (*p* = 0.005) of SB 242084 increased trials to criterion in reversal two. SB 242084 had no effect on trials to criterion in discrimination learning (*F*_3,29_ = 0.761, *p* = 0.525).

On incorrect responses to criterion (Fig. 1f), there was significant main effects of test phase (*F*_2,58_ = 184.884, *p* < 0.0001) and dose (*F*_3,29_ = 5.448, *p* = 0.004) and a significant dose × phase interaction (*F*_6,58_ = 2.783, *p* = 0.019). 0.1 mg/kg (*p* = 0.023) and 1 mg/kg (*p* = 0.004) of SB 242084 increased incorrect responses in reversal one. 0.5 mg/kg (*p* = 0.014) 1 mg/kg (*p* = 0.003) of SB 242084 increased incorrect responses in reversal two. SB 242084 had no effect on incorrect responses in discrimination learning (*F*_3,29_ = 0.772, *p* = 0.519).

For previous CS+ errors (Fig. 1 g), there were significant main effects of phase (*F*_1,29_ = 168.7, *p* < 0.0001) and dose (*F*_3,29_ = 4.12, *p* = 0.015) but no dose × phase interaction (*F*_3,29_ = 2.361, *p* = 0.092). Animals made more previous CS+ errors in reversal 2 than in reversal 1. 0.5 mg/kg (*p* = 0.013) and 1 mg/kg (*p* = 0.005) of SB 242084 increased previous CS+ errors relative to vehicle treated controls.

For constant CS− errors (Fig. 1 h), there were significant main effects of phase (*F*_1,29_ = 66.497, *p* < 0.0001) and dose (*F*_3,29_ = 3.086, *p* = 0.043) but no significant dose × phase interaction (*F*_3,29_ = 2.791, *p* = 0.058). Animals made more constant CS− errors in reversal 2 than reversal 1. 0.5 mg/kg (*p* = 0.024) and 1 mg/kg (*p* = 0.01) of SB 242084 increased constant CS− errors relative to vehicle treated controls.

For early errors (Fig. 2d), there was a significant effect of reversal phase (*F*_1,29_ = 27.937, *p* < 0.0001), dose (F_3,29_ = 3.075, *p* = 0.043) and dose × reversal phase interaction (*F*_3,29_ = 3.342, *p* = 0.033). 0.1 mg/kg (*p* = 0.044) and 1 mg/kg (*p* = 0.006) of SB 242084 decreased early errors in reversal 1. On late errors (Fig. 2c), there was a significant effect of reversal phase (*F*_1,29_ = 104.286, *p* < 0.0001) and dose (*F*_3,29_ = 6.406, *p* = 0.002) with SB 242084 increasing late errors relative to vehicle treated controls. Moreover, there was a significant effect of dose on trials (*F*_3,29_ = 3.922, *p* = 0.018), incorrect responses (*F*_3,29_ = 3.279, *p* = 0.035), late errors (*F*_3,29_ = 3.189, *p* = 0.038) but not early errors (F_3,29_ = 2.535, *p* = 0.076) in the drug-free reversal 3.

In sum, SB 242084 dose dependently decreased early errors but increased late errors in reversal learning without affecting discrimination learning. SB 242084 also decreased stimulius response latencies.

### Experiment 3—SB 242084 and 2-stimulus visual reversal learning

**Fig. 3:**
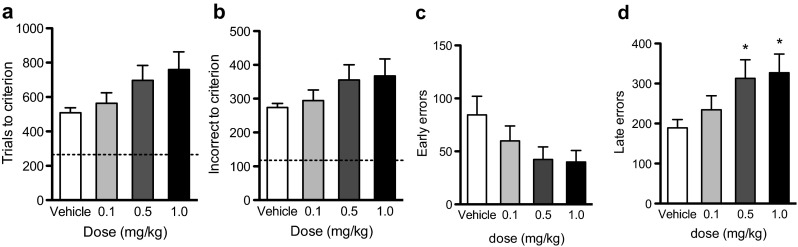
Dose-dependent effects of SB 242084 on 2-stimulus reversal learning in the rat. **a** Trials to criterion. No effect of dose but a significant dose-linear effect. **b** Incorrect responses. No effect of dose. **c** Early errors. No effect of dose but a significant dose linear effect. **d** Late errors. Significant effect of dose as well as a dose linear effect. *Broken line* represents mean discrimination learning performance. *Asterisks* denote *p* < 0.05 vs. vehicle (*p < 0.05)

In 2-stimulus reversal learning (Fig. 3a–d) there were trends for SB 242084 to decrease the number of early errors but increase the number of late errors causing an overall increase in the number of trials and incorrect responses to criterion. SB 242084 significantly decreased stimulus response times and pellet retrieval latencies.

There was no significant effect of SB 242084 trials to criterion (Fig 3a; *F*_3,42_ = 2.426, *p* = 0.079), incorrect responses to criterion (Fig. 3b; *F*_3,42_ = 1.485, *p* = 0.232) omissions to criterion (F_3,42_ = 0.901, *p* = 0.449) or early errors (Fig. 3c; *F*_3,42_ = 2.206, *p* = 0.102). However, there were significant dose-linear effects of SB 242084, with higher doses increasing trials to criterion (*F*_1,42_ = 7.123, *p* = 0.011) and decreasing early errors (*F*_1,42_ = 5.823, *p* = 0.020). There was a significant effect of SB 242084 on late errors to criterion (Fig. 3d; *F*_3,42_ = 2.916, *p* = 0.045) with 0.5 and 1 mg/kg of SB 242084 increasing the number of errors in the late phase of learning (*p* ≤ 0.025).

SB 242084 also decreased response and pellet retrieval latencies (Table 4). There was a significant main effect of dose on stimuli response latencies (*F*_3,42_ = 5.719, *p* = 0.002), with all doses decreasing the time taken to respond. There was also a significant effect of dose on pellet retrieval latencies (*F*_3,42_ = 3.831 *p* = 0.016), with 0.5 and 1 mg/kg (*p* ≤ 0.023) of SB 242084 decreasing the time taken for collection of pellet reward.

**Table 4:** Mean touch-screen response and reinforcer retrieval latencies in seconds (±SEM) in experiment 3

	Discrimination^a^		Reversal	
Dose	Response	Retrieval	Response	Retrieval
Vehicle	3.35 ± 0.16	2.05 ± 0.17	2.89 ± 0.11	1.72 ± 0.11
0.1	3.00 ± 0.20	2.04 ± 0.21	2.28 ± 0.17**	1.49 ± 0.10
0.5	3.12 ± 0.17	2.00 ± 0.14	2.27 ± 0.13**	1.43 ± 0.07*
1. 0	3.03 ± 0.15	1.93 ± 0.14	2.24 ± 0.11**	1.31 ± 0.06**

Asterisks denote significant difference from vehicle (*p < 0.05; **p < 0.01)

^a^Drug free

### Experiment 4—development of a touchscreen serial visual reversal task and validation using intra-OFC baclofen/muscimol infusions

In preliminary experiments (data not shown), optimal parameters for the serial visual reversal task (e.g. robust perseverative responding across multiple reversals) were established and defined as a running criterion of 24 correct/30 trials and two (drug-free) retention sessions between reversals. These parameters were then used throughout experiments 4 and 5.

**Fig. 4:**
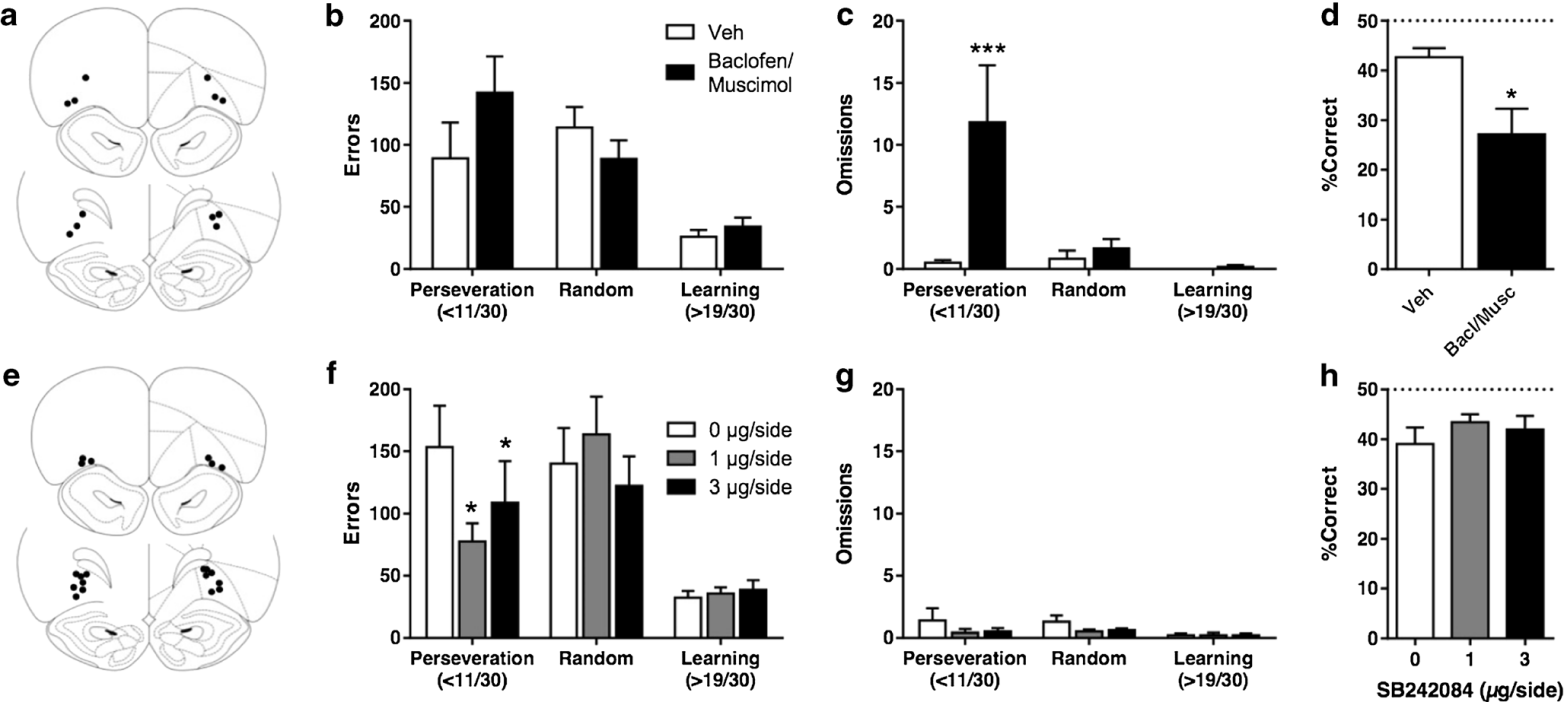
Effects of pharmacological inactivation and intracerebral SB 242084 infusions on the touchscreen serial visual reversal task. **a** Histological validation of baclofen/muscimol cohort. A total of six rats had cannulae targeting the lateral OFC and received all infusions. **b** Effect of pharmacological inactivation on total trials. **c** Effect of pharmacological inactivation on omissions. Note that omissions are only possible after the rats have initiated a trial by responding at the ‘start box’ (see Supplementary Fig. 1). **d** Effect of pharmacological inactivation on percent correct responses during the first reversal session (**e**) histological validation of OFC SB 242084 cohort. A total of 10 rats had cannulae targeting the lateral OFC and received all infusions. **f** Effect of intra-OFC SB 242084 infusions on total trials across phases. **g** Effect of intra-OFC SB 242084 infusions on omissions. **h** Effect of intra-OFC SB 242084 on percent correct during the first reversal session. *Asterisks* denote *p* < 0.05 vs. vehicle (*p < 0.05; ***p < 0.001)

Six rats had intact cannulae throughout the study and injector tips inside the lateral OFC. Infusions of a baclofen/muscimol cocktail into the lateral OFC impaired early performance on the serial visual reversal task (Fig. 4a–d). After baclofen/muscimol inactivation of the OFC, two-way repeated measures ANOVA revealed a significant main effect of phase on trials (*F*_2,10_ = 5.507, *p* = 0.024) and a non-significant effect of drug (*F*_1,5_ = 5.382, *p* = 0.068); no interaction was noted (*F*_2,10_ = 1.988, *p* > 0.10). Planned comparisons in the perseverative phase showed a trend for the effect of OFC inactivation on perseverative trials (*p* = 0.094). On the number of incorrect responses (Fig. 4b), there was a main effect of phase (*F*_2,10_ = 12.54, *p* = 0.0019) and a non-significant trend for effect of drug (*F*_1,5_ = 5.296, *p* = 0.070) but no interaction (*F*_2,10_ = 2.058, *p* > 0.10). In a planned comparison of incorrect responses during the perseverative phase, a non-significant trend of drug was observed (*p* = 0.077).

On omissions (Fig. 4c), there was an effect of phase (*F*_2,10_ = 7.426, *p* = 0.011), drug (*F*_1,5_ = 11.15, *p* = 0.021) and a phase × drug interaction (*F*_2,10_ = 8.133, *p* = 0.008). Post hoc tests revealed that baclofen/muscimol infusions increased the number of omissions during the perseverative phase (*p* = 0.0002), but had no effect during the random or learning phases. Baclofen/muscimol infusions reduced percent correct responses on the first day of reversal (Fig. 4d; *t*_5_ = 3.126, *p* = 0.026). No significant effect was noted on either response latency (paired *t* test, *t*_5_ = 1.711; *p* > 0.10) or latency to collect rewards (paired *t* test, *t*_5_ < 1, *p* > 0.10; Table 5).

**Table 5:** Mean response latency and reinforcer retrieval latency in seconds (±SEM) after micro-infusions in experiments 4 and 5

	Baclofen/muscimol			SB 242084	
Dose	Response	Retrieval	Dose	Response	Retrieval
Vehicle	0.72 ± 0.03	1.17 ± 0.20	Vehicle	1.10 ± 0.12	0.79 ± 0.10
1 mM	0.85 ± 0.08	1.22 ± 0.15	1 μg/side	1.14 ± 0.10	0.86 ± 0.15
			3 μg/side	1.04 ± 0.09	0.85 ± 0.10

No significant effect of drug

### Experiment 5—intra-OFC infusions of SB 242084 in the serial visual reversal task

Ten rats had intact cannulae throughout the study and injector tips inside the lateral OFC. SB 242084 locally infused into the OFC-improved performance during the early, perseverative phase (Fig. 4e–h). In a two-way repeated-measures ANOVA, there was a significant main effect of phase on trials (*F*_2,18_ = 9.03, *p* = 0.0019) but no effect of drug (*F*_2,18_ = 1.04, *p* > 0.10) or phase × drug interaction (*F*_4,36_ = 1.49, *p* > 0.10). However, planned comparisons in the perseverative phase showed a reduced number of trials at 1 μg/side (*p* = 0.046) and a trend at 3 μg/side (*p* = 0.070). On the number of incorrect responses (Fig. 4f), there was a main effect of phase (*F*_2,18_ = 13.28, *p* = 0.0003) but no effect of drug (*F*_2,18_ = 1.215, *p* > 0.10) or interaction (*F*_4,36_ = 1.811, *p* > 0.10). In a planned comparison of incorrect responses during the perseverative phase, SB 242084 reduced the number of errors at 1 μg/side (*p* = 0.022) and at 3 μg/side (*p* = 0.042).

On omissions (Fig. 4 g), there was a main effect of phase (*F*_2,18_ = 4.49, *p* = 0.026) but no effect of drug (*F*_2,18_ = 1.83, *p* > 0.10) and no phase × drug interaction (*F*_4,36_ < 1, *p* > 0.10). Planned comparisons in the perseverative phaserevealed no significant effect of SB 242084 infusions, but a trend at 1 μg/side (*p* = 0.059). Intra-OFC infusions of SB 242084 did not affect percent correct responses on the first day of reversal (Fig. 4 h; F_2,27_ < 1, *p* > 0.10). Likewise, no significant effect was noted on either response latency (*F*_1.809,16.28_ = 1.107, *p* > 0.10) or latency to collect rewards (*F*_1.694,13.55_ < 1, *p* > 0.10); see Table 5.

## Discussion

Systemic 5-HT_2C_R antagonism by SB 242084 improved performance in the early stages of touchscreen-based visual reversal learning in the rat. This apparently cognitive-enhancing dose-dependent effect was observed across two separate tasks and reproduced following direct microinfusions of SB 242084 into the rat OFC, which has been previously implicated in reversal learning.

Surprisingly, in view of these early improvements in reversal learning and previously published data (Boulougouris et al. 2008; Nilsson et al. 2012), systemic SB 242084 additionally impaired performances in the later stages of reversal learning, thus causing an overall decrement in performance. This effect was reproduced across paradigms and observed cross-site in the laboratories of both academic and industrial partners of the NEWMEDS collaboration. However, the detrimental effects were not observed after OFC infusion suggesting that the early improvement in reversal learning may depend on a selective reduction in perseverative responding mediated by 5-HT_2C_Rs in the OFC.

This study has provided an innovative new suite of methods for assessing visual reversal learning in the rat. We adapted a 3-stimulus version previously used in primates to further analyse the nature of the reversal-learning effects (experiments 1 and 2). We also introduced a novel 2-stimulus version suitable for within-subject neuropharmacological investigations that was validated to be dependent on OFC circuitry via baclofen/muscimol inactivation (experiment 4). The discussion will focus on the desirability of replicating findings across academic laboratories and the industrial setting and evaluating the methodological innovations reported in this study as well as the role of 5-HT in modulating neural circuitry underlying reversal learning and its possible clinical implications.

### Academic-industrial cross-site replication

There is an urgent need for replication of behavioural findings following either genetic (Crabbe and Wahlsten 1999) or pharmacological (Insel et al. 2013) manipulations in experimental animals. We have addressed this objective by introducing rodents (Bussey et al. 2012), which via the intermediaries of computer-controlled touchscreen tasks have translational relevance to human tests such as the CANTAB battery (Robbins et al. 1994; Robbins et al. 1998). Visual reversal learning has been shown to be readily translatable across species from mouse to rat to non-human primate to human participants (Keeler and Robbins 2012). Hitherto, the human and non-human primate tests of reversal learning have used computer-controlled touchscreen methods to study visual reversal whereas rodent versions have often employed olfactory or other non-visual modalities. However, Chudasama and Robbins (2003) did use similar methods to those in primates when investigating the effects of OFC manipulations on visual reversal learning in the rat. The present study has developed this methodology further by employing carefully chosen visual stimuli that optimise rapid learning and also enable serial reversal learning to be investigated sometimes within the same test session. This new protocol has been distributed among several laboratories in the NEWMEDS consortium, and in this report, we show that effects of pharmacological manipulations can be readily replicated and extended by this form of academic-industrial collaboration. The use of novel touchscreen tasks reported here adds further translational value to previous results and indicates that 5-HT_2C_R mechanisms play an important role in visual reversal learning.

### 5-HT_2C_R antagonism improves early reversal learning: neural substrates

The novel observation that 5-HT_2C_R antagonism can improve aspects of visual reversal learning is in agreement with previous studies on spatial reversal. Thus, using a spatial, left-right serial reversal paradigm in an operant chamber, systemic 5-HT_2C_R antagonism also decreased early errors (Boulougouris et al. 2008) and attenuated subchronic PCP-induced reversal-learning impairments in rats (McLean et al. 2009). Decreased activity at the 5-HT_2C_R through constitutive or pharmacological inactivation also improved aspects of reversal learning in mice (Nilsson et al. 2012; Nilsson et al. 2013). The improved reversal learning following OFC 5-HT_2C_R antagonism is in contrasts with the impaired reversal learning associated with decreased 5-HT action as a whole through OFC 5-HT-depletion in marmoset monkeys (Clarke et al. 2007) or as a function of interindividual variations in OFC 5-HT markers in the rat (Barlow et al. 2015).

An obvious interpretation of the reduction in early errors by SB 242084 is that it reduces perseverative responding (Boulougouris et al. 2008). However, in the present study, we found no evidence of specific amelioration of stimulus-perseveration responding using the novel 3-stimulus reversal paradigm which has been suggested to distinguish between effects on perseveration or more general learning (Jentsch et al. 2002). Instead, SB 242084 had similar effects on both constant CS− errors and previous CS+ errors. This could be in line with the previous observation of Nilsson et al. (2012) that systemic 5-HT_2C_R antagonism facilitated ‘learned non-reward’ while the perseverative repetition of a previously reinforced choice was unaffected. In contrast, the impairment associated with 5,7-DHT lesion of the OFC in marmoset monkeys was selectively displayed when the animals were tested for stimulus perseveration (Clarke et al. 2007).

In agreement with previous reports (Boulougouris and Robbins 2010), the current data show that the ability of SB 242084 to improve aspects of reversal learning is related to its effects in the OFC. The 5-HT_2C_R appears to have an inhibitory function on neuronal activity within the prefrontal cortex; these receptors are present on GABAergic, primarily parvalbumin-containing, interneurons (Liu et al. 2007). Furthermore, micro-iontophoretic application of non-selective 5-HT_2C_R agonists suppresses firing rates in the OFC or mPFC (El Mansari and Blier 1997; Bergqvist et al. 1999; Zghoul and Blier 2003). Increased OFC activity has previously been linked with improved reversal learning (O'Doherty et al. 2001), and thus, the observed improved reversal performance may result from SB 242084 potentiating such activity in this area. In further support of this view, 5-HT_2C_R antagonism elevates DA-dialysate levels in the PFC (Millan et al. 1998; Gobert and Millan 1999; Gobert et al. 2000) and loss of 5-HT_2C_R function can cause glutamatergic supersensitivity at OFC AMPA receptors (Rueter et al. 2000). Potentiation of AMPA-receptor transmission can have pro-cognitive effects, including PFC-specific enhancements of LTP-formation (Black 2005), improved reversal learning in the bowl-digging procedure (Woolley et al. 2009) and attenuation of the attentional set-shifting deficits produced by subchronic PCP (Broberg et al. 2009). Thus, the observed improvement following OFC-specific SB 242084 infusions may be related to decreased 5-HT_2C_R function potentiating glutamatergic and dopaminergic signalling within the OFC. One caveat in this context is that we did not observe a dose-dependent effect of SB 242084 in the current experiment. This might be related to the higher dose range employed here; it can be speculated that full receptor occupancy is reached already at the 1 μg/side dose or even that off-target effects counteract the main effect on 5-HT_2C_R in the OFC at the 3 μg/side dose, although the affinity of SB 242084 is more than 100-fold higher for 5-HT_2C_R than for other receptors (Kennett et al. 1997).

### 5-HT_2C_R impairs late reversal performance: systemic effects

Systemic SB 242084 was also found to impair overall reversal learning by increasing the number of late errors to criterion. This effect appears to be independent from the effect of 5-HT_2C_R antagonism on early reversal learning, as it did not occur following central microinfusions of the drug. Previous studies of the 5-HT_2C_R in reversal learning using a variety of paradigms have reported inconsistent results on late errors (Boulougouris et al. 2008; Boulougouris and Robbins 2010; Nilsson et al. 2012; Pennanen et al. 2013; Nilsson et al. 2013). Nevertheless, it is clear that any of the possible ‘cognitive-enhancing’ effects of the 5-HT_2C_R antagonist when systemically administered have to be interpreted in the context of additional possible impairments, which may even lead to overall deficits. The interesting issue is how this additional, detrimental effect may arise. Chudasama and Robbins (2003) found that there were at least two influences on the overall attainment of successful reversal performance, an inhibitory action on perseverative responding mediated by the rodent lateral OFC and a separate effect on new associative learning mediated by the medial PFC. Thus, it is possible that the 5-HT_2C_R antagonist has dissociable effects on the OFC and medial PFC, impairing the function of the latter. Clearly, systemic administration of the antagonist would affect both regions to produce these opposed effects on reversal-learning performance. This could be approached in future experiments by investigating reversal learning after 5-HT_2C_R blockade in the medial PFC through SB 242084 microinfusions.

An alternative possibility is that the 5-HT_2C_R antagonist exerts its behavioural effects on reversal-learning performance indirectly through other actions. SB 242084 has previously been shown to exacerbate premature, ‘impulsive’ responding, for example in the 5-choice serial reaction time task (5-CSRTT; Fletcher et al. 2007) even after profound 5-HT depletion (Winstanley et al. 2004). Thus, it was previously proposed that 5-HT_2C_R antagonism has opposite effects on compulsive behaviour in the form of reversal learning and impulsive behaviour (Robbins and Crockett 2010). However, it is also possible that these apparently contrasting effects to improve early reversal learning and also impair late reversal performance all arise as a consequence of the enhanced impulsivity produced by the 5-HT_2C_R antagonist. Thus, elevated impulsivity could interfere with efficient choice behaviour by causing rapid, random, responding that increases late errors to criterion. This interpretation is supported by the observation that in addition to the increased errors responding following systemic SB 242084 administration is also faster in terms of both response latencies and pellet retrieval times. As well as indicating possible impulsivity, these additional behavioural effects might reflect motivational influences, for example, on feeding at the level of the hypothalamus (Heisler et al. 2003).

It should also be considered that a similar effect on impulsivity/motivation may account for the early improvement in reversal learning. Thus, for example, if a tendency to impulsive behaviour interferes with perseverative responding, this may indirectly lead to an elimination of early errors. However, against this interpretation are the observations that (i) although systemic SB 242084 consistently speeded responding, it had no detrimental effect on initial discrimination learning prior to reversal and (ii) inspection of discrimination performance during early reversal (the initial 20 trials) fails to show any immediate ‘beneficial’ effect of systemic SB 242084 and (iii) speeded responding, i.e. reduced response latencies, was not observed after central administration of SB 242084 despite its cognitive-enhancing effect.

### Towards a reversal-learning test battery for rodents

We here report the development of two novel touchscreen reversal tasks for the rat: a 3-stimulus paradigm that allows the selective investigation of stimulus-perseveration during reversal and a 2-stimulus serial reversal paradigm that allows within-subject systemic and local pharmacological investigations. These two paradigms will be useful in different experimental contexts or, as here, in conjunction.

The addition of a third stimulus to the discrimination task stems from previous work in vervet monkeys (Jentsch et al. 2002) and allows the separate investigation of stimulus-perseveration errors towards the previous CS+ and errors of an explorative or general nature towards the unchanged, ‘constant’ CS−. This approach may be particularly valuable when pharmacological or genetic manipulations can be expected to involve or affect inflexible responses to a stimulus that was previously rewarded, such as after 5-HT depletion of the OFC (Clarke et al. 2007) or perseveration induced by cocaine (Jentsch et al. 2002).

The serial reversal paradigm, in contrast, was developed to study the neuropharmacology of reversal learning using local drug micro-infusions. In previously established visual discrimination protocols, rodents require a large number of sessions to reach criterion on visual reversal learning in the touchscreen setting (Mar et al. 2013), making local infusions throughout the reversal phase difficult. This obstacle to neuropharmacological investigations was previously addressed by micro-infusions on a subset of sessions, i.e. during early, intermediate and late stages (Brigman et al. 2013). Here, we instead developed a paradigm that allows the animals to reach criterion in a brief period of time (typically three sessions) and demonstrate that the optimised protocol robustly produces perseverative responding across multiple reversals. Importantly, performance on this paradigm engages the lateral OFC; previous serial reversal paradigms using various modalities have yielded inconsistent results, with lesions producing impaired performance (Rygula et al. 2010), no effect (Boulougouris and Robbins 2009) or either improved or impaired performance depending on the length of training between reversals (Riceberg and Shapiro 2012).

## Conclusion

The present, cross-site study has shown dose-dependent improvements in early reversal learning produced by the 5-HT_2C_R antagonist SB 242084 either following systemic or central OFC administration which may have translational relevance to reversal-learning impairments associated with neuropsychiatric disorders such as schizophrenia or OCD. However, we have additionally shown that systemic (but not central) administration of the drug leads to additional effects including speeded responding and impaired overall reversal performance, thus highlighting potential problems in its clinical utility.

## Electronic supplementary material

ESM 1 (DOCX 189 kb)
