# Supplementary material for: The role of 5-HT2C receptors in touchscreen visual reversal learning in the rat: a cross-site study
Source: Psychopharmacology (Berl). 2015 May 26;232(21-22):4017–31. doi: 10.1007/s00213-015-3963-5 (PMC4600472; doi:10.1007/s00213-015-3963-5)
Supplement: Supplementary file 1 — (DOCX 189 kb) [file 213_2015_3963_MOESM1_ESM.docx]

Supplementary material

The role of 5-HT_2C_ receptors in touchscreen visual reversal learning in the rat: a cross-site study

Alsiö, J.^1,2,3^, Nilsson, SRO.^1,2^, Gastambide, F.^4^, Wang, RAH.^1,2^, Dam, S.A.^1,2^, Mar, AC.^1,2^, Tricklebank, M.^4^, & Robbins, TW.^1,2^

^1^Department of Psychology

University of Cambridge

Cambridge, CB2 3EB, UK

^2^Behavioural and Clinical Neuroscience Institute,

University of Cambridge

Cambridge, CB2 3EB, UK

^3^Department of Neuroscience

Unit of Functional Neurobiology

University of Uppsala

Uppsala, SE-75124 Sweden

^4^Lilly Centre for Cognitive Neuroscience,

Eli Lilly & Co. Ltd, Erl Wood Manor,

Windlesham, GU20 6PH, UK.

Communicating author: [ja476@cam.ac.uk](mailto:ja476@cam.ac.uk), Tel +44 1223 766157


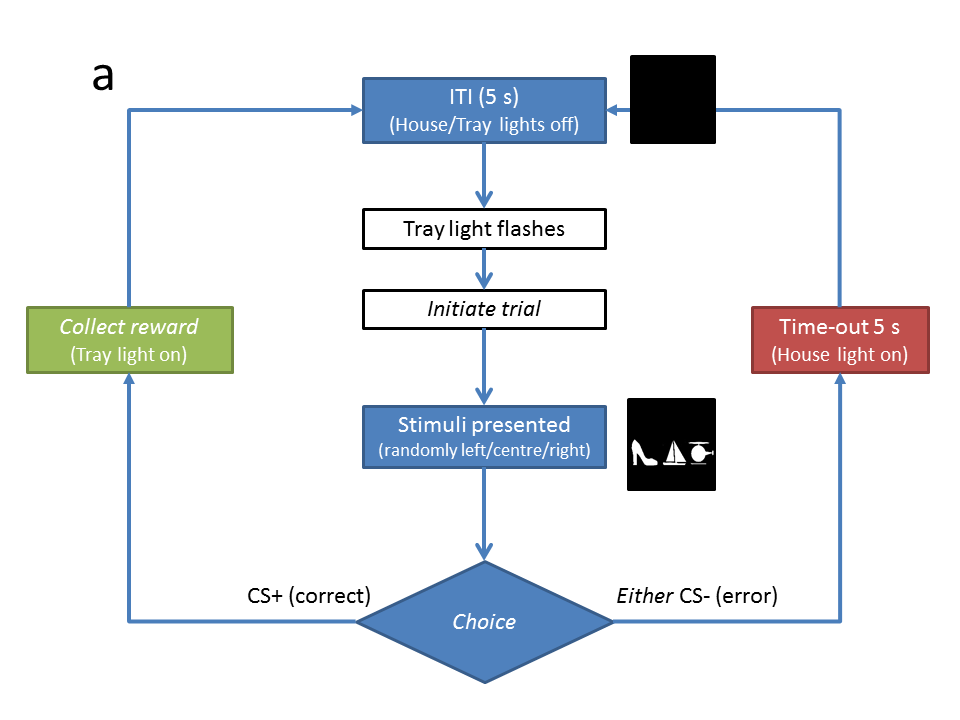


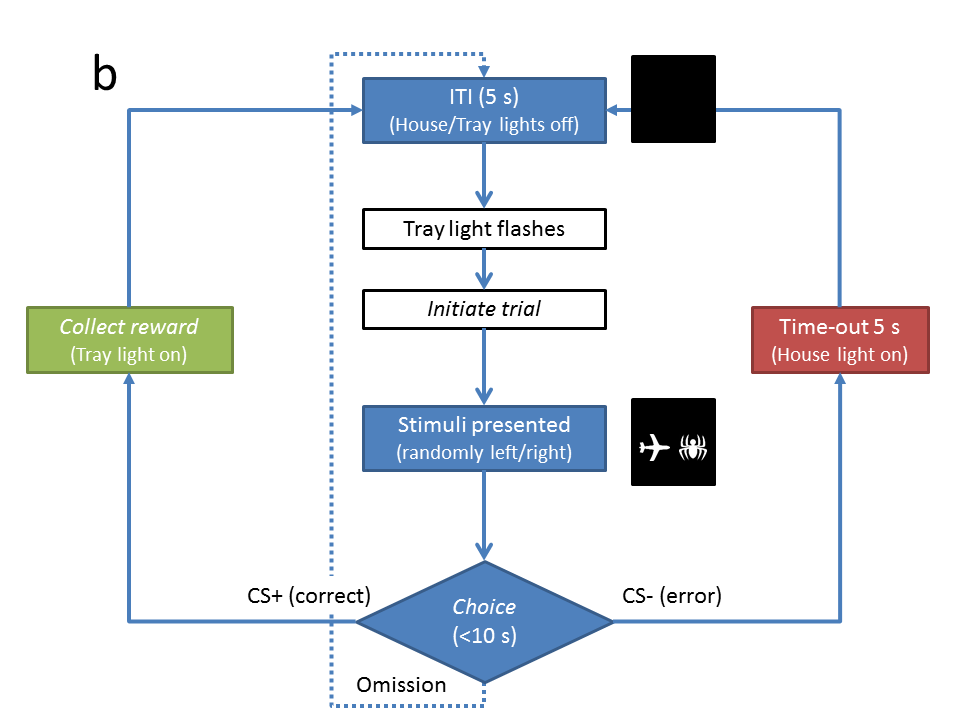


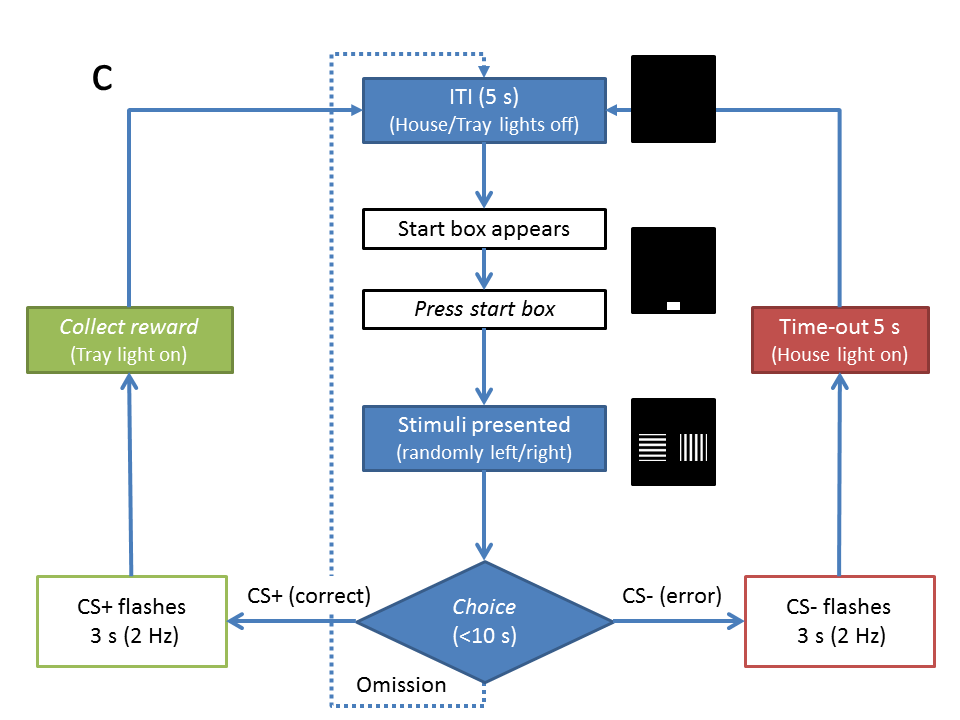


**Supplementary Figure 1.** Flowchart depiction of the visual discrimination and reversal tasks. a) 3-stimulus reversal task (Experiments 1 and 2); b) 2-stimulus reversal task (Experiment 3); c) 2-stimulus serial visual reversal task (Experiments 4 and 5). In the 2-stimulus serial reversal task, the start box was 4cm wide and 3cm tall. Reward delivery was coupled with a short tone in the 3-stimulus and 2-stimulus reversal tasks but not the serial reversal task. ****

**Supplementary Figure 2.** Performance over the 300 first trials (30-trial bins) after microinfusions into the OFC. a) Experiment 4: Impaired early performance after pharmacological inactivation of the OFC with baclofen/muscimol (N = 6); ***p < .001 vs. vehicle treatment. b) Experiment 5: Improved early performance after SB242084 infusions into the OFC (N=9); *p < .05 SB242084 at 1 µg/side vs. vehicle, ^§^p < .05 SB242084 at 3 µg/side vs. vehicle.

**SUPPLEMENTARY METHODS AND MATERIALS**

Apparatus

The experiments used 32 operant chambers (Med Associates, Georgia, VT, USA; 30 cm × 39 cm × 29 cm) placed in sound-attenuating wooden boxes with fans for the purpose of ventilation and masking external noise; 16 boxes were at the industrial partner and 16 at the academic partner. In each box, a central magazine connected to an external pellet dispenser delivering 45mg sucrose pellet (TestDiet 5TUL; Sandown Scientific, Middlesex, UK). A houselight was located near the ceiling directly above the magazine. The opposite side of the chamber contained a touch-sensitive screen which was covered with a 3-hole (Experiment 1 and 2) or a 2-hole (Experiment 3) Perspex mask creating three or two response-windows (8 × 8 cm) through which the animals could touch the screen. The response-windows were spaced 2.5 cm apart in the 2-hole mask and 1.5 cm apart in the 3-hole mask. At the academic partner, task schedules were developed and implemented by A.C.M. using Visual Basic 2010. At the industrial partner, schedules were implemented by in-house software.

Experiment 1-3: 2- and 3-stimulus reversal learning

*Pretraining stage 1.* Animals initially received a single 45min session of Pavlovian and instrumental training. The session began with the delivery of a 45mg sucrose pellet paired with a 1s tone and the onset of the magazine-light. The tone and onset of magazine-light were coupled with delivery of pellet reward throughout all phases of both experiments. Collection of pellet reward caused a photobeam to break and resulted in the offset of the magazine-light and the presentation of a white square stimulus (6cm × 6cm) positioned 1cm from the bottom of the screen in three (Experiments 1-2) or two (Experiment 3) possible response windows. The white square was presented equal number of times in each response-window but the same response-window was never used for more than two consecutive trials to help prevent the formation of location biases. A nosepoke on the white-square caused the stimulus to disappear and led to pellet delivery. If the animal failed to touch the white-square within 30s, the white-square disappeared, a pellet was delivered and the trial was recorded as an omission. When the animal collected the pellet reward the magazine-light was extinguished and a new trial was initiated.

*Pretraining stage 2*. As Stage 1, a session began with the delivery of a pellet reward coupled with a 1s tone and the onset of the magazine-light. A nosepoke in the magazine turned the magazine-light off and caused the presentation of the white square stimulus in one of the response windows position 2cm from the bottom of the screen. Touching the stimulus resulted in pellet delivery, and a new trial started when pellet reward was collected. The criterion was ≥100 correct responses in a 45min session.

*Pretraining stage 3*. A 5s ITI was now introduced. Collection of pellet reward caused the magazine light to turn off and the initiation of a 5s ITI when the chamber remained dark. When the ITI had elapsed, the magazine-light began flashing at a rate of 1Hz to distinguish it from the steady magazine-light associated with pellet delivery. A nosepoke in the magazine turned the flashing light off and initiated a new trial. The session ended after 45min or 100 trials, and the criterion was ≥100 correct trials in 45min.

*Pretraining stage 4.* Similar to Stage 3, however, responses to unlit response window(s) were made more undesirable. If the animal touched an unlit response-window, the stimulus was immediately removed, the houselight was illuminated for a 5s time-out period, and an incorrect response was recorded. Following the 5s time-out period, the houselight was extinguished and the 5s ITI was initiated prior to commencement of the next trial (the same ITI period remained following correct responses). After the ITI had elapsed, the magazine-light began flashing at 1Hz and new trial started when the animal nosepoked in the magazine. The session ended after 45min or 100 trials, and criterion was ≥75 correct responses in a 45min session.

Experiment 4-5: Touchscreen serial visual reversal

*Pretraining stage 1.* Rats responded at a single white box at the bottom centre of the screen for sucrose reward pellets during 60-minute daily sessions until they reliably received 100 pellets in one day. Across sessions, the size of the white box was reduced until it measured approximately 3 x 4cm.

*Pretraining stage 2.* Touching the white ‘start’ box was not reinforced, but led to the presentation of a single stimulus on the screen (horizontal or vertical bars) to the left or right in a pseudo-random order. Responding at this stimulus was reinforced with a reward pellet, whereas responding to the blank side was signalled as incorrect by the illumination of the house light for a 5s time-out period. After the rats had reached ≥80% correct touches, the position of the stimuli was raised approximately 5cm on the screen in order to avoid accidental touches. The single stimulus presented was horizontal or vertical bars on alternate days. After the rats again had reached ≥80% correct touches on both stimuli, visual discrimination training ensued.

**SUPPLEMENTARY RESULTS**

Experiment 4: Effects of baclofen/muscimol micro-infusions on performance across the first 300 trials

To investigate whether the effects of pharmacological inactivation of the OFC was dependent on the a priori-defined phases (Perseveration, Random, Learning), we analysed the performance after baclofen/muscimol infusions in 30-trial bins over the first 300 trials (Supplementary Fig. 2a). In agreement with the trend observed in the Perseveration phase as well as with the effect observed on Omissions and %correct on the first day of reversal, there was a main effect of drug (F_1,5_ = 12.97, p = .016) and bin (F_9,45_ = 12.22, p < .0001) as well as a significant drug × bin interaction (F_9,75_ = 2.540, p = .019); post-hoc analyses revealed a significant effect of the pharmacological inactivation at the 3^rd^ 30-trial bin after reversal (p < .001).

Experiment 5: Effects of SB242084 micro-infusions on performance across the first 300 trials

Similarly to above, we analysed the performance after SB242084 infusions in 30-trial bins over the first 300 trials (Supplementary Fig. 2b). There was a main effect of bin (F_9,72_ = 15.45) and drug (F_2,16_ = 4.55, p = .027) but no drug × bin interaction (F_18,144_ < 1, p > .10). Post-hoc analysis showed that 1 µg/side significantly improved performance at the 1^st^ (p = .010), 7^th^ (p = .022), and 8^th^ 30-trial bin (p = .013), whereas 3 µg/side significantly improved performance at the 6^th^ 30-trial bin (p = .013). One animal reached criterion within 300 trials after vehicle infusions and was thus excluded from this analysis.
